# Carrying both *COL1A2* and *FBN2* gene heterozygous mutations results in a severe skeletal clinical phenotype: an affected family

**DOI:** 10.1186/s12920-022-01296-8

**Published:** 2022-07-08

**Authors:** Jing Chen, Qinqin Xiang, Xiao Xiao, Bocheng Xu, Hanbing Xie, He Wang, Mei Yang, Shanling Liu

**Affiliations:** 1 Department of Obstetrics and Gynecology, West China Second University Hospital, Sichuan University, Chengdu, 610041 Sichuan China; 2 Key Laboratory of Birth Defects and Related Diseases of Women and Children (Sichuan University), Ministry of Education, No. 20, Section 3, Renminnan Road, Chengdu, 610041 Sichuan China

**Keywords:** Osteogenesis imperfecta, Congenital contractural arachnodactyly syndrome, *COL1A2*, *FBN2*, Synergistic effect, Whole-exome sequencing

## Abstract

**Background:**

Osteogenesis imperfecta (OI) is the most common monogenic disease of the skeletal system and is usually caused by mutations in the *COL1A1* or *COL1A2* genes. Congenital contractural arachnodactyly syndrome (CCA) is an autosomal dominant hereditary disease of connective tissue. To date, the *FBN2* gene is the only gene reported to cause CCA. Researchers found that *COL1A2* and *FBN2* are both involved in the extracellular matrix organization pathway. These findings suggest that these two genes play an important role in a similar mechanism and may trigger a synergistic effect.

**Methods:**

Trio-whole-exome sequencing (Trio-WES) was performed to analyse the underlying genetic cause of a proband with OI in a Chinese family. Sanger sequencing was used to validate the mutations in 3 members of the family with OI with varying degrees of severity of skeletal abnormalities and the members with no clinical signs.

**Result:**

A c.3304G > C mutation in the *COL1A2* gene (p.Gly1102Arg) and a novel c.4108G > T mutation in the *FBN2* gene (p.Glu1370*) were detected in the proband, an affected member of the family. The affected individuals with both mutations present a more severe phenotype, while affected individuals present a milder phenotype if only the mutation in *COL1A2* is detected (c.3304G > C). The unaffected individual in this family did not have any mutations in the *COL1A2* gene or *FBN2* gene.

**Conclusion:**

Our study is the first clinical report to indicate that patients carrying concomitant mutations in both the *COL1A2* and *FBN2* genes may present with more severe skeletal abnormalities. Furthermore, our study suggests the possibility of synergistic effects between the *COL1A2* and *FBN2* genes.

**Supplementary Information:**

The online version contains supplementary material available at 10.1186/s12920-022-01296-8.

## Background

Osteogenesis imperfecta is a genetic disorder of increased bone fragility, low bone mass, and other connective-tissue manifestations. In the majority of cases, osteogenesis imperfecta is caused by mutations in *COL1A1* or *COL1A2*, which are genes that encode the two collagen type I alpha chains [1, 2]. The incidence of osteogenesis imperfecta in newborns in China is approximately 1/15,000–1/77,000 [3]. OI has a variable phenotype, even among patients in the same family [4–6]. According to clinical presentation, radiographic findings, and family history, *COL1A1/2* osteogenesis imperfecta (*COL1A1/2-*OI) is mainly classified into four types (I-IV) and is usually inherited in autosomal dominant (AD) pattern [4, 7]. OI-I has the mildest phenotype [2]. Patients with OI-II usually die during the perinatal period [8], and OI-III patients have recurrent fractures and severe bone deformation. Patients with mild to moderate bone deformities and short stature are classified as OI-IV; the severity of OI types increases in the order OI-I < OI-IV < OI-III < OI-II [2]. With the constant increase in the number of identified mutations in *COL1A1*, *COL1A2* or other genes, genotype–phenotype correlation have become increasingly pertinent [1]. Contractural arachnodactyly syndrome (CCA) is a rare autosomal dominant connective tissue disease that is characterized by arachnodactyly, contractures of major joints and progressive scoliosis, and its clinical features partially overlap with the phenotype of Marfan syndrome (MFS) [9–11]. The alpha2 chain of the type I collagen (*COL1A2*) and fibrillin-2 (*FBN2*) genes, which are the cause of these two diseases, respectively, are members of the extracellular matrix pathway. This implies that these two genes may be functionally related and may trigger a synergistic effect.

In this report, we first describe a Chinese family in which the proband and his affected father carried heterozygous mutations in the *COL1A2* gene (OMIM: 120160; c.3304G > C; p.Gly1102Arg) and *FBN2* gene (OMIM: 612570; c.4108G > T; p.Glu1370*) that caused a significantly more severe phenotype of OI. The proband’s elder sister, who had a milder clinical phenotype, only carried a mutation in the *COL1A2* gene (OMIM: 120160; c.3304G > C; p.Gly1102Arg).

## Method

### Clinical data

**Fig. 1:**
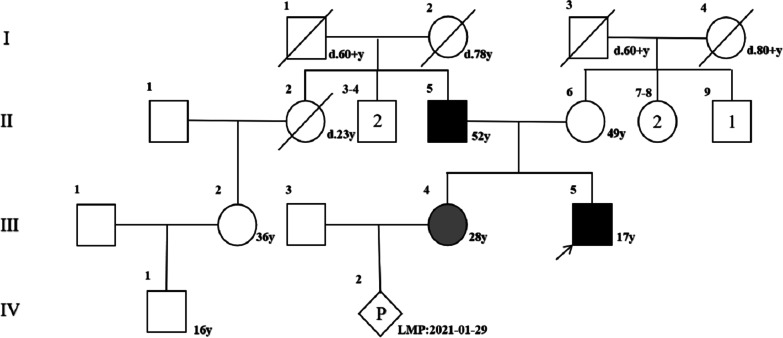
Pedigree of the proband’s family.Pedigree of a four-generation family with recurrent fractures and bone deformity. Generations are shown as I–IV. Squares indicate male, and circles indicate female. Empty symbols indicate unaffected individuals and filled symbols indicate affected individuals.Deceased individuals are indicated by a slash (/), the arrow shows the proband

A 28-year-old female (III4) who was three months pregnant sought genetic counselling and prenatal diagnosis for the foetus in the current pregnancy at West China Second University Hospital, Sichuan University (Chengdu, China). The woman informed the doctor that her family members had skeletal diseases of variable severity. The proband (III5, the pregnant woman’s brother) was a 17-year-old male who had over sixteen fractures after trauma, of which the long bones of the limbs and ribs were usually involved. At the time of examination, he showed short stature, slender upper limbs, slender fingers, severe joint contractures, significant muscle atrophy and severe skeletal deformities and was unable to walk. Their father (II5) had symptoms similar to those of the proband but could walk slowly on crutches. The pregnant woman (III4) had the mildest phenotype in this family. She only had three fractures to date. She presented with only an old fracture of the left elbow and a mild limitation of dorsoextension. Her appearance and height were normal. The pedigree of this family is shown in Fig. 1.

Trio-whole-exome sequencing (Trio-WES) was performed to analyse the underlying genetic cause of the family. Suspected mutations detected by next-generation sequencing (NGS) were validated by Sanger sequencing.

### DNA extraction

Total genomic DNA was extracted from whole blood from the proband and his family members using a DNeasy Blood & Tissue DNA kit (Qiagen, Hilden, Germany) according to the manufacturer’s instructions.

### Exome sequencing and bioinformatic analysis pipeline

To detect the variants carried by the proband, the Nano WES Human Exome V1 (Berry Genomics) was used to capture the sequences. Then, the enriched library was sequenced on a Nova seq 6000 with 150 paired-end reads. The reads were mapped to a human reference genome (hg38) with BWA (v0.7.15). Variant calling was performed by Verita Trekker (v1.2.0.2). During the analysis of the data, we selected the variants if their minor allele frequencies (MAF) were < 0.05 in the 1000 Genomes Project (1000G) (http://browser.1000genomes.org), Exome Aggregation Consortium (ExAC) (http://exac.broadinstitute.org/), and gnomAD (http://gnomad.broadinstitute.org/). SNVs with a minor allele frequency (MAF) ≥ 1% for a dominant inheritance pattern were excluded. For pathogenicity prediction, CADD (https://cadd.gs.washington.edu), SIFT (http://sift.jcvi.org), PolyPhen-2 (http://genetics.bwh.harvard.edu/pph2), and Rare Exome Variant Ensemble Learner (REVEL) (https://sites.google.com/site/revelgenomics/) were used. To select disease-causing variants, we referred to the information from the OMIM database (http://www.omim.org), ClinVar database (http://www.ncbi.nlm.nih.gov/clinvar) and Human Gene Mutation Database (http://www.hgmd.org). SNVs were classified into five categories, pathogenic (P), likely pathogenic (LP), uncertain significance (VUS), likely benign (LB) and benign (B), according to the guidelines of the American College of Medical Genetics (ACMG) [12]. ACMG is based on population data, computational and predictive data, functional data, segregation data, de novo data, and allelic data. The detailed process for identifying candidate variants is shown in Additional file 1: Fig. S1.

### Sanger sequencing

To validate the disease-causing variants selected, Sanger sequencing was performed using specific PCR primers designed with Primer Premier 5. The sequences of the *FBN2* primers used were *FBN2*-F: 5′-GCAAACTCACCAATACACTT-3′ and *FBN2*-R: 5′-CTCCATACGGTTGCATCTT-3′. The sequences of the *COL1A2* primers used were *COL1A2*-F: 5′-GAACATGCTTCCGTGTGA-3′ and *COL1A2*-R: 5′-CATCAACTTCATAGTCCTTGG-3′. PCR products were sequenced using an ABI 3500 Genetic Analyser (Thermo Fisher Scientific) for *COL1A2* c.3304G > C and *FBN2* c.4108G > T.

## Results

### Heterozygous mutations of the COL1A2 and FBN2 genes were identified in a family with a skeletal clinical phenotype

**Fig. 2:**
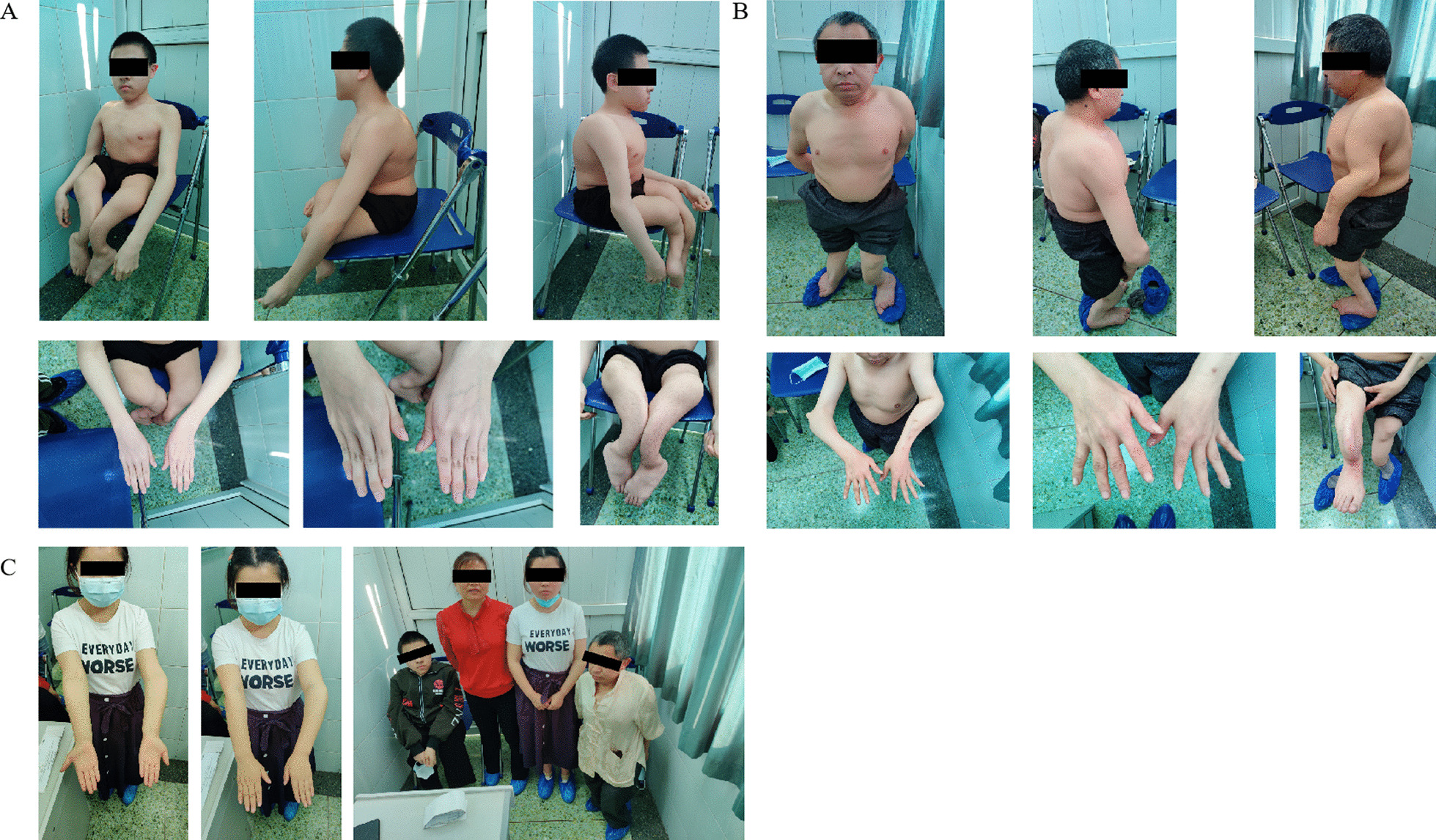
The clinical symptoms of proband’family. **A** The proband showed short stature, barrel chest, kyphosis, slender fingers and other skeletal deformities. **B** The proband’s father showed similar symptoms to the proband; **C** The proband's sister had the mildest skeletal system abnormality,and from left to right is the proband, the proband's mother, the proband’s sister and the proband's father

**Fig. 3:**
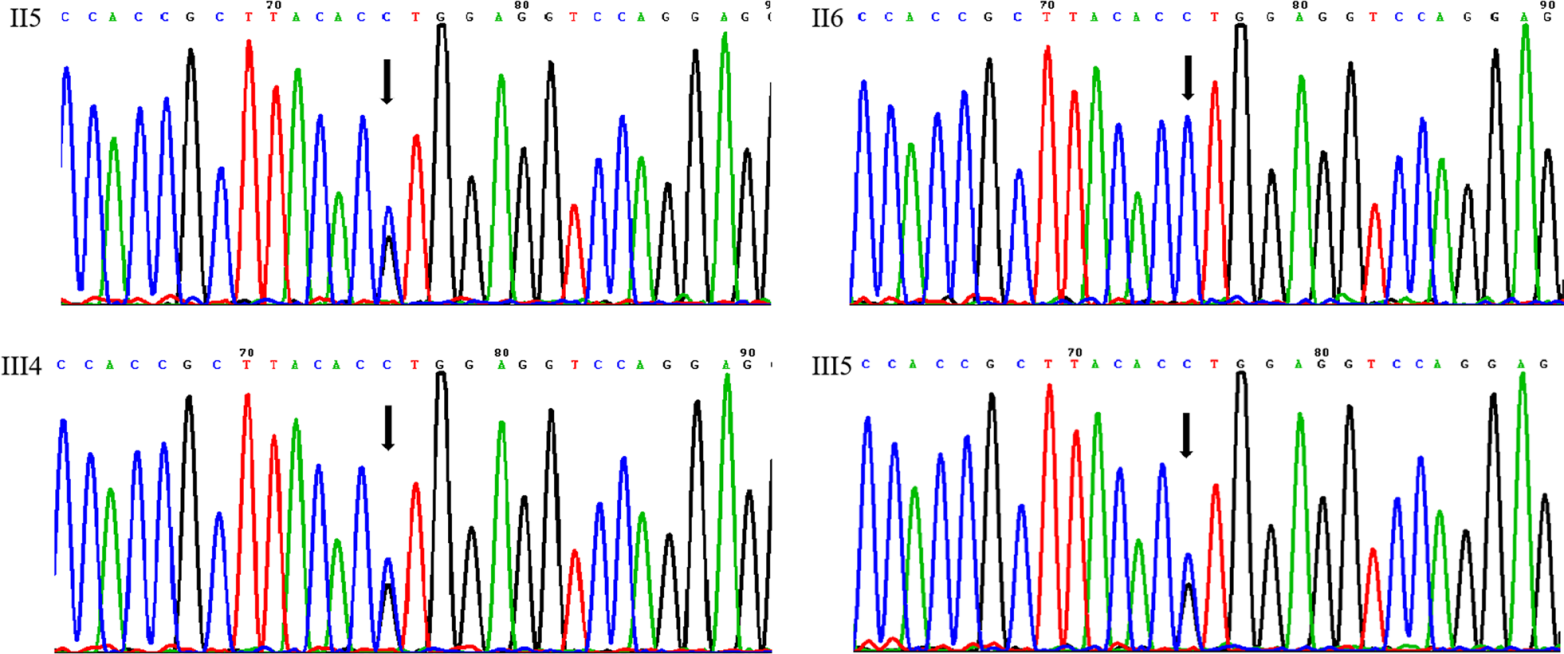
Sanger sequencing chromatograms of the II5, II6, III4 and III5 (*COL1A2*, c.3304G > C). The *COL1A2* missense mutation was detected in all affected individuals (II5, III4 and III5) but not in unaffected members (II6) by Sanger sequencing. The black arrows indicate the point of mutation (G > C)

**Fig. 4:**
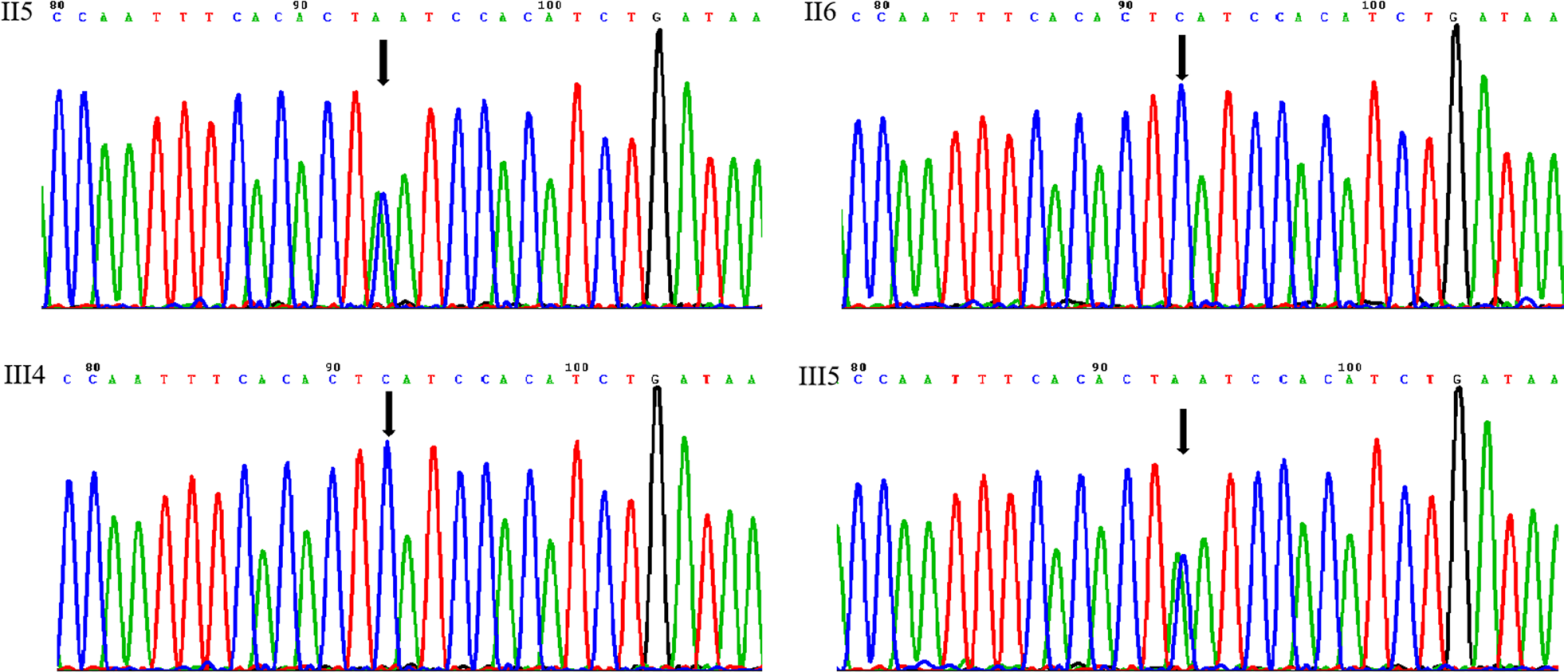
Sanger sequencing chromatograms of the II5, II6, III4 and III5(*FBN2*, c.4108G > T) The *FBN2* nonsense mutation was detected in severer affected individuals (II5, III5) but not in clinically less affected member (III4) or unaffected member (II6) by Sanger sequencing. The black arrows indicate the point of mutation (G > T)

A missense mutation in exon 49 of *COL1A2* (NM_000089.4; c.3304G > C; p.Gly1102Arg) was detected in the proband (III5); this mutation was inherited from the patient’s affected father (II5). Additionally, a nonsense mutation in exon 32 of *FBN2* (NM_001999.4; c.4108G > T; p.Glu1370*) was also detected in the proband; this mutation was also inherited paternally. In other words, both the proband (III5) and his father (II5) had a severe skeletal clinical phenotype and carried concomitant mutations in both the *COL1A2* and *FBN2* genes. The presence of the mutations was further validated by Sanger sequencing for the proband (III5) and his father (II5) (Figs. 3, 4). However, no mutations were detected in the unaffected mother (II6) (Figs. 3, 4). No other pathogenic or likely pathogenic variants related to the skeletal disorders were detected in the mother. The mutations and clinical data of the patients in the family are shown in Table 1 and Fig. 2. The presence of the mutations was further validated by Sanger sequencing in the affected elder sister (III4), who presented with a milder phenotype. Only the mutation in *COL1A2* (c.3304G > C) was detected in the affected elder sister (III4) (Figs. 3, 4).

**Table 1:** Mutations and clinical features of individuals included in the study

	II5	III5	III4	II6
Gene mutation	*COL1A2* c.3304G > C *FBN2* c.4108G > T	*COL1A2* c.3304G > C *FBN2* c.4108G > T	*COL1A2* c.3304G > C	None
*Phenotype*				
Short stature	**+++ **(1.1 m*)	**+++ **(1.1 m)	**− **(1.56 m)	**− **(1.54 m)
Sclera	**−**	**−**	**−**	**−**
Recurrent fractures	**+++ **(≥ 10 times)	**+++ **(≥ 16 times)	**+ **(3 times)	**−**
Scoliosis	**+**	**+**	**−**	**−**
Acromacria	**+**	**+**	**−**	**−**
Joint contracture	**++**	**++**	**−**	**−**
Muscle hypotrophy	**++**	**++**	**−**	**−**
Intellectual development or other systems	**−**	**−**	**−**	**−**

Symbol +/− indicates whether there is a clinical phenotype, the number of + indicates the severity of the phenotype

*m: meter

### In silico* analysis*

**Fig. 5:**
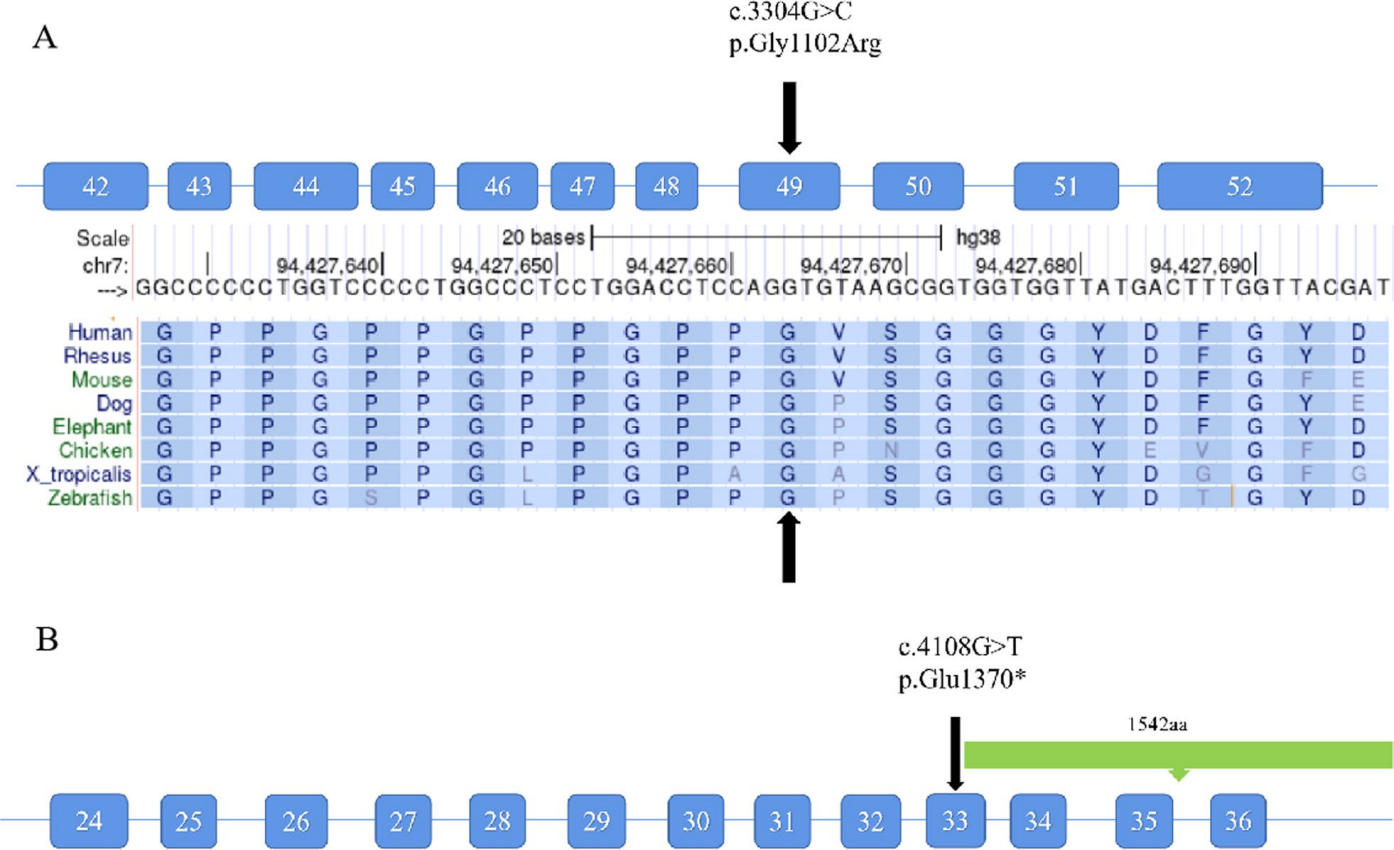
**A** The missense mutation (c.3304G > C) in the *COL1A2* gene (NM_000089.4) results in replacement of glycine by a highly conserved arginine (p.G1102R); **B** The nonsense mutation (c.4108G > T) in the FBN2 gene (NM_001999.4) results in the premature termination of transcription (p.E1370*)

The mutation (c.3304G > C) in *COL1A2* results in replacement of a highly conserved glycine (polar amino acid) by arginine (basic amino acid). In silico analysis of the *COL1A2* mutation (p.Gly1102Arg) indicated that this substitution is disease causing and deleterious, as determined by CADD_Phred, SIFT_pred, Polyphen2_HVAR_pred, and REVEL. A change in the same codon that results in a p.Gly1102Arg substitution had been previously reported as a *COL1A2* mutation associated with Osteogenesis imperfecta IV [17]. In addition, multiple sequence alignment of *COL1A2* from different species showed the evolutionary conservation of the glycine residue at position 1102 (Fig. 5).

The nonsense mutation (c.4108G > T) in *FBN2* (NM_001999.4) results in a premature termination codon at amino acid position 1370 (p.Glu1370*). The probability of loss-of-function intolerance (pLI = 0.99) of this mutation was greater than 0.9, revealing that this nonsense mutation was intolerant to loss-of-function (LoF). Furthermore, the NMD_predict forecasted that this nonsense mutation might generate nonmediated decay (NMD).

According to the guidelines of ACMG, the mutation (c.3304G > C) in *COL1A2* and the nonsense mutation (c.4108G > T) in *FBN2* were both classified as likely pathogenic (LP) and are considered to be the cause of the clinical manifestations in this family.

## Discussion

The *COL1A2* gene encodes the pro-alpha2 chain of type I collagen, whose triple helix comprises two alpha1 chains and one alpha2 chain [13]. Mutations in *COL1A2* are associated with osteogenesis imperfecta types II-IV, Ehlers–Danlos syndrome, and idiopathic osteoporosis. Wenstrup et al. identified the same heterozygous mutation (c.3304G > C) of *COL1A2* in a family with an autosomal dominant form of mild-moderate osteogenesis imperfecta. The affected members of this family had bone fragility, short stature and dentinogenesis imperfecta but had no clinical manifestations of slender fingers and severe joint contractures [14]. In this report, the proband, his affected father and less affected sister all carried the same mutation (c.3304G > C) of the *COL1A2* gene, and the proband and his father had slender fingers and severe joint contracture. This phenotype is not consistent with the phenotypes of osteogenesis imperfecta or Ehlers–Danlos syndrome and cannot be explained by genetic heterogeneity. It is likely that the clinical variation among the affected and less affected members of this family results from synergistic effects with other genes involved in matrix production.

*FBN2* is an extracellular matrix gene that encodes fibrillin 2. It is a component of connective tissue microfibrils and may be involved in elastic fibre assembly [15, 16]. Mutations in *FBN2* are associated with CCA. CCA is a connective tissue disease characterized by arachnodactyly, contractures of major joints and progressive scoliosis [17]. These phenotypes are similar to the slender fingers and joint contractures observed in the proband and his father. Genetic analysis identified a novel heterozygous *FBN2* nonsense mutation (c.4108G > T; p.Glu1370*) in the proband and his affected father. However, this mutation was not found in his sister, who demonstrated a milder phenotype. These observations suggest a synergistic effect of these mutant alleles of two related but distinct genes, *COL1A2* and *FBN2*, and this finding provides evidence for a digenic form of skeletal disorders.

In this report, a heterozygous mutation in *COL1A2* combined with another heterozygous mutation in *FBN2* simultaneously aggravates the skeletal clinical phenotype in individuals. The Reactome signal pathway database (https://reactome.org) and previous studies show that both *COL1A2* and *FBN2* are involved in the extracellular matrix organization pathway [11, 18], suggesting that both genes play an important role in the assembly and degradation of the extracellular matrix (ECM). It is well known that the extracellular matrix can send signals to cells to direct or regulate the transcription of certain mRNAs [19–21]. Therefore, we suspect that a mutation in one of the two genes, *COL1A2* and *FBN2*, may affect the expression level of the other gene, but this requires further research.

Compared to monogenic inheritance, digenic inheritance does not follow the rules of Mendelian inheritance, so it is often underdiagnosed due to the difficulty of verifying the true synergistic effect. The members of the extracellular matrix structural constituent, *COL4A3, COL4A4* and *COL4A5*, have been reported to be consistent with digenic inheritance and, together, lead to the occurrence of Alport syndrome [22–24]. *COL1A2* and *FBN2* are also members of the extracellular matrix pathway. Thus, these two genes may be functionally related and may also have a synergistic effect.

Large-scale research projects in genetic diseases have indicated that massively parallel whole-genome/whole-exome sequencing can reveal a large number of new genes or new alleles that otherwise would be undetected by traditional sequencing methodologies [25]. At present, whole-exome sequencing based on next-generation sequencing technology is one of the most effective tools for the diagnosis of genetic diseases. Our results provide accurate genetic information for targeted treatment, genetic counselling and subsequent prenatal diagnosis for the patients in this family.

## Conclusion

In conclusion, we successfully identified a mutation in exon 49 of *COL1A2* (c.3304G > C; p.Gly1102Arg) and another novel heterozygous *FBN2* mutation (c.4108G > T; p.Glu1370*) in this family. The novel variant expands the spectrum of mutations in the *FBN2* gene. Furthermore, this is the first clinical report to identify patients carrying coexisting mutations in both the *COL1A2* and *FBN2* genes that contribute to more severe skeletal abnormalities. Furthermore, our segregation analysis indicated the possibility of synergistic effects between the *COL1A2* and *FBN2* genes.

## Supplementary Information

**Additional file 1**. **Figure S1.** The detailed process for identifying candidate variants.

## Data Availability

For the considerations about the security of human genetic resources and the confdentiality of participant, the data is not publicly available, but can be obtained from the corresponding author on reasonable request.

## Abbreviations

OI: Osteogenesis imperfecta
CCA: Contractural arachnodactyly syndrome
Trio-WES: Trio-whole-exome sequencing
AD: Autosomal dominant
MFS: Marfan syndrome
NGS: Next-generation sequencing
P: Pathogenic
LP: Likely pathogenic
VUS: Uncertain significance
LB: Likely benign
B: Benign
ACMG: American College of Medical Genetics
LoF: Loss-of-function
NMD: Non-mediated decay
*COL1A1*: Alpha1 chain of type I collagen
*COL1A2*: Alpha2 chain of type I collagen
ECM: Extracellular matrix
*FBN1*: Fibrillin-1
*FBN2*: Fibrillin-2
