# Supplementary figures and images for: Carrying both COL1A2 and FBN2 gene heterozygous mutations results in a severe skeletal clinical phenotype: an affected family
Source: BMC Med Genomics. 2022 Jul 8;15:154. doi: 10.1186/s12920-022-01296-8 (PMC9270787; doi:10.1186/s12920-022-01296-8)

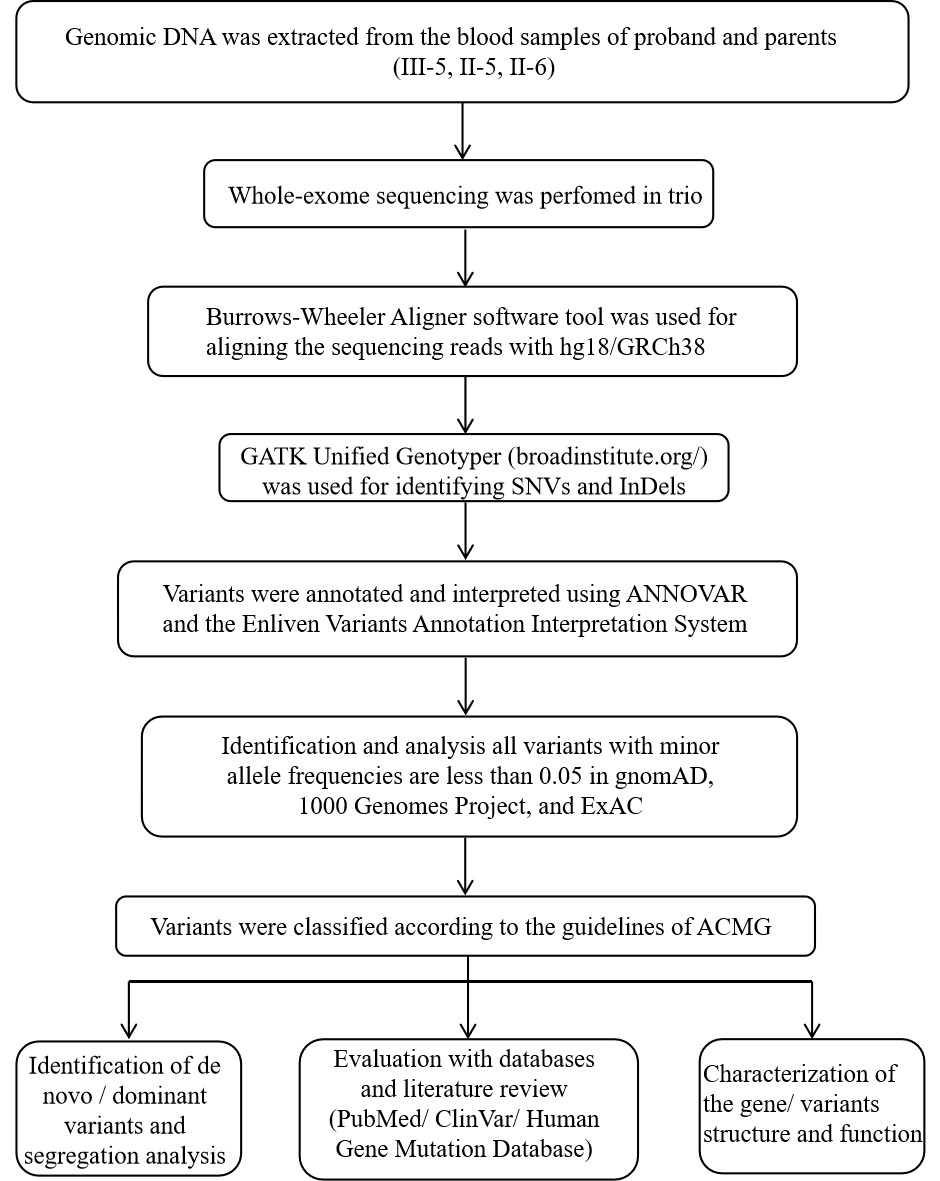

Supplement: Supplementary file 1 — Additional file 1. Figure S1. The detailed process for identifying candidate variants. [file 12920_2022_1296_MOESM1_ESM.jpg]
